# Probiotics for the Prevention of Vaginal Infections: A Systematic Review

**DOI:** 10.7759/cureus.64473

**Published:** 2024-07-13

**Authors:** Andrea M Zuñiga Vinueza

**Affiliations:** 1 School of Medicine, Universidad Catolica de Santiago de Guayaquil, Guayaquil, ECU

**Keywords:** systematic review, randomized controlled trials, vulvovaginal candidiasis, bacterial vaginosis, vaginal health, probiotics

## Abstract

Probiotics, particularly *Lactobacillus *strains, have been proposed as an alternative or adjunct therapy for bacterial vaginosis (BV) and vulvovaginal candidiasis (VVC) due to their potential to restore a healthy vaginal microbiota. This systematic review evaluated 11 randomized controlled trials with a Jadad score greater than three, indicating high-quality studies based on criteria such as randomization, blinding, and dropout rates. The review demonstrated significant improvements in clinical outcomes and vaginal microbiota restoration. However, variability in results highlights the need for further research.

## Introduction and background

Bacterial vaginosis (BV) and vulvovaginal candidiasis (VVC) are the two most prevalent types of vaginal infection [1-5]. Each presents with a distinct set of clinical symptoms, and they are considered distinct entities with distinct etiologies and pathogenesis [1]. Mixed infections involving simultaneous BV and VVC are rare, with limited understanding of their relationship and interactions. A study of women in Argentina with recurrent vulvovaginal candidiasis (RVVC) found that 35% were associated with BV, and 33.2% had an intermediate vaginal microbiota [1].

Bacterial vaginosis, marked by a reduction in *Lactobacillus *spp. and overgrowth of anaerobic bacteria, affects 20%-50% of women globally [2]. Vulvovaginal candidiasis, primarily caused by *Candida albicans*, affects approximately 75% of women at least once in their lifetime, with 40%-50% experiencing recurrent episodes [3]. Conventional treatments for BV and VVC, including antibiotics and antifungals, often fail to prevent recurrence owing to persistent biofilms and emerging drug resistance [4]. Probiotics, particularly *Lactobacillus *strains, have been proposed as a promising alternative or adjunct therapy for BV and VVC because of their ability to restore and maintain healthy vaginal microbiota [5]. In Ecuador, probiotics are primarily available as oral, over-the-counter medications. This highlights the necessity of reviewing the availability of new formulations and alternative uses to enhance their effectiveness in different populations and conditions.

Previous studies have examined the use of probiotics to treat or alleviate BV and VVC through oral and vaginal administration. Although these findings appear promising, the effectiveness and mechanisms of probiotics in this context remain largely unexplored and inconsistent. A systematic review is necessary to consolidate the evidence, identify gaps, and clarify the role of probiotics in managing BV and VVC. This review aimed to evaluate the effectiveness of probiotics in preventing BV and VVC, to identify the most effective strains and administration methods, and to provide evidence-based clinical recommendations. Given the high prevalence and recurrence of these infections, understanding the potential of probiotics could enhance management strategies and improve women's reproductive health outcomes [1-5].

## Review

Materials and methods

Study Design

This systematic review was designed to evaluate the effectiveness of probiotics used solely as an intervention to prevent infections in various clinical trials. The inclusion criteria for this review mandated that the studies focus on infection prevention rather than treatment and provide comprehensive methodological details for quality assessment using the Jadad scale.

Search Strategy

A comprehensive literature review was carried out in June 2024 by utilizing the PubMed and Web of Science (WOS) databases, incorporating the keywords "probiotics," "bacterial vaginosis," and "candidiasis, vulvovaginal." The search results were further refined by applying filters to only publications with free full text, randomized controlled trials (RCTs), and those published within the past five years in either English or Spanish. This approach was taken to ensure that the most current and relevant studies were included, reflecting current research trends and advancements in the field. The reference lists of relevant articles were also examined to identify additional studies. The decision to limit the search to the past five years was made to guarantee that the studies included were the most recent and relevant, reflecting current research trends and advancements in the field.

A total of 114 studies were retrieved through the comprehensive search: 23 articles from PubMed and 91 from WOS. The abstracts and titles of these articles were reviewed for eligibility based on the inclusion and exclusion criteria following the PRISMA guidelines.

Inclusion and Exclusion Criteria

The inclusion and exclusion criteria are presented in Table 1.

**Table 1:** Inclusion and exclusion criteria RCTs: randomized controlled trials

Criteria Type	Criterion
Inclusion	Studies published in English or Spanish
Inclusion	RCTs that assessed the preventive effects of probiotics
Inclusion	Conducted on human participants
Inclusion	Probiotics used alone without adjunctive antimicrobial therapy
Exclusion	Studies that focused on treatment rather than prevention
Exclusion	Non-randomized or observational studies
Exclusion	Studies that did not provide clear data on probiotic strains and dosages
Exclusion	Phase 1 or Phase 2 studies, study protocols, and studies not providing outcome data

Data Extraction

**Figure 1:**
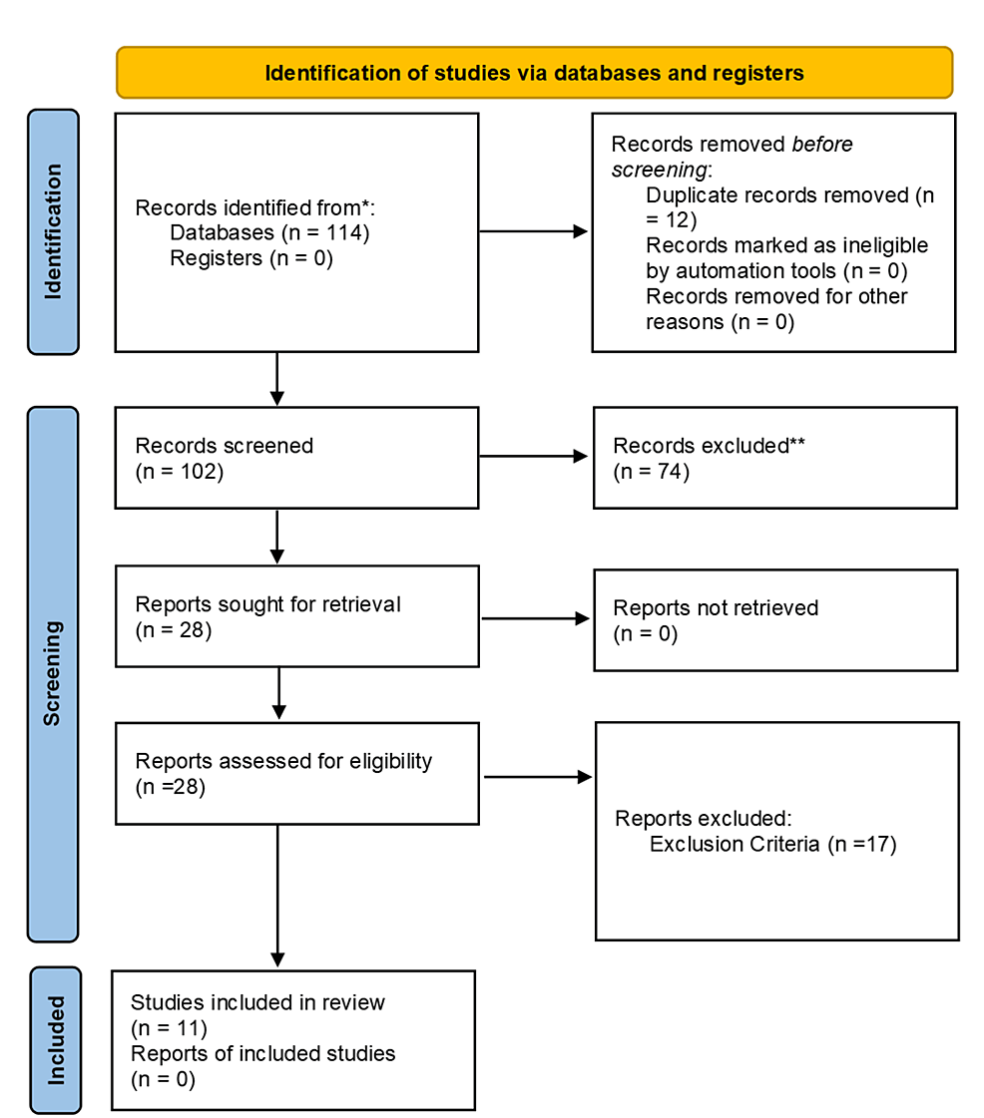
A PRISMA flow diagram outlining the study selection process This flow diagram details the selection process for the systematic review, including the identification, screening, eligibility, and inclusion of studies. The initial search yielded 114 records from PubMed and the Web of Science (WOS). After removing duplicates, 102 records were screened for relevance, and 74 were excluded. Full-text assessments were conducted on 28 articles, resulting in 11 studies meeting the inclusion criteria for the final review. *Sources: PubMed and Web of Science **Exclusion reasons: Protocol studies, Phase 1 or 2 studies, studies focusing on or using antimicrobial agents or other drugs. PRISMA: Preferred Reporting Items for Systematic Reviews and Meta-Analyses

The information extracted from eligible studies included the following: title, authors, publication year, study design, sample size, population characteristics, and inclusion and exclusion criteria. Two reviewers independently extracted the information. Intervention details, including the type of probiotics used, dosage, and duration of the intervention, were also extracted. Outcomes were categorized as primary and secondary outcomes, and documented results and conclusions were included (Figure 1).

Quality Assessment

In addition to the Jadad scale, which evaluates randomization, blinding, and withdrawals, several other quality aspects are crucial for the comprehensive assessment of study reliability. These aspects include allocation concealment, adherence to intervention protocols, and handling of missing data [6, 7].

The Jadad scale, also known as the Oxford quality scoring system, consists of three items: randomization, blinding, and withdrawals/dropouts. Each component earned a score from 0 to two, resulting in a maximum score of five. A total score of 0 to two indicates low quality, while three to five indicates high quality [6]. 

Adhering to intervention protocols is crucial for maintaining consistency with the study design, which involves monitoring and documenting participants' compliance with the probiotic regimen and any deviations [7]. Handling the missing data is critical. Effective strategies include intention-to-treat analysis, imputation methods, and thorough documentation of reasons for dropout or non-compliance, ensuring robust study conclusions [7].

Data Synthesis

Research articles that fulfilled the inclusion criteria and attained a minimum score of three on the Jadad scale were considered for qualitative synthesis. A narrative synthesis strategy was utilized to summarize the results, concentrating on the effectiveness of probiotics in preventing infections and unfavorable occurrences.

Results

In the systematic review process, a comprehensive search was conducted across multiple databases. The search strategy yielded 23 articles from PubMed, 91 articles from the WOS, and 0 articles from the Cochrane database. After applying the inclusion and exclusion criteria, 11 studies met the criteria for inclusion in the systematic review. These studies were selected because they specifically focused on the preventive effects of probiotics without the adjunctive use of antibiotics or other pharmaceuticals. The studies included were all RCTs with a Jadad score of three or higher, ensuring high methodological quality (Table 2).

**Table 2:** Characteristics of the included studies on probiotics for vaginal health RCT: randomized controlled trial; BV: bacterial vaginosis; VVC: vulvovaginal candidiasis; PPROM: preterm pre-labor rupture of membranes; PROM: pre-labor rupture of membranes; IL: interleukin; VAS: visual analog scale; VT: vaginal tablets

Authors	Year	Methods	Interventions and comparators	Main results	Conclusions	Randomization	Randomization method	Double-blind	Double-blind method	Withdrawals and dropouts	Jadad score
Mändar R, et al. [8]	2023	RCT, 182 women (89 BV, 93 VVC)	Probiotics (oral/vaginal) vs. placebo	Significant improvement in BV and VVC symptoms, increase in *Lactobacillus*	Probiotics effective for BV and VVC, administrable orally and vaginally	Yes	Double-blind randomization	Yes	Indistinguishable placebo	Not specified	4
Yang S, et al. [9]	2020	RCT, 86 pregnant women	Probiotics vs. placebo	No difference in vaginal microbiota diversity between groups	Probiotics do not adversely affect vaginal microbiota during pregnancy	Yes	Double-blind randomization	Yes	Indistinguishable placebo	Not specified	4
Ang XY, et al. [10]	2023	RCT, 78 pregnant women	Probiotics (SynForU-HerCare) vs. placebo	Improvement in vaginal and gastrointestinal microbiota in the probiotics group	Probiotics prevent adverse changes in microbiota during VC	Yes	Double-blind randomization	Yes	Indistinguishable placebo	Not specified	4
Park SH, et al. [11]	2023	RCT, 101 women	MED-01 vs. placebo	Significant reduction in the Nugent score in the probiotics group	MED-01 is effective for treating BV, improves vaginal microbiota	Yes	Double-blind randomization	Yes	Indistinguishable placebo	Not specified	4
Jepsen IE, et al. [12]	2022	RCT, 74 women	*Lactobacillus *vs. placebo	No significant improvement in vaginal microbiota with probiotics	Probiotics do not significantly modulate vaginal microbiota before fertility treatments	Yes	Double-blind randomization	Yes	Indistinguishable placebo	Not specified	4
Armstrong, Eric; et al. [13]	2022	Randomized, placebo-controlled, double-blind trial	LACTIN-V vs. placebo	Lower concentrations of IL-1 alpha and soluble E-cadherin	LACTIN-V reduced genital inflammation and epithelial barrier disruption	Yes	Described and appropriate	Yes	Described and appropriate	Yes	5
Bangar, Sampada; et al. [14]	2023	Randomized, placebo-controlled, double-blind trial	*Lactobacillus *VT vs. placebo	No significant difference in BV recurrence between groups	*Lactobacillus *VT was acceptable and safe but did not show additional benefit over metronidazole	Yes	Described and appropriate	Yes	Described and appropriate	Yes	5
Koirala, Ranjan; et al. [15]	2023	Randomized, placebo-controlled, double-blind crossover trial	*Lactobacillus paracasei* LPC-S01 vs. placebo	Reduction in the relative abundance of Gardnerella spp.	Potential positive effect on the vaginal microbial ecosystem	Yes	Described and appropriate	Yes	Described and appropriate	Yes	5
Vaccalluzzo, Amanda; et al. [16]	2023	Randomized, placebo-controlled, double-blind trial	*Lacticaseibacillus rhamnosus* TOM 22.8 vs. placebo	Restoration of the physiological pH and reduction of potentially pathogenic bacteria	Effective strategy for the treatment of vaginal dysbiosis	Yes	Described and appropriate	Yes	Described and appropriate	Yes	5
Vanda, Raziyeh, et al. [17]	2023	Randomized, placebo-controlled, double-blind trial	Oral probiotic vs. placebo	Reduction in PPROM and PROM	Oral probiotics can reduce complications such as PPROM and PROM	Yes	Described and appropriate	Yes	Described and appropriate	Yes	5
Vivekanandan et al. [18]	2023	Randomized, placebo-controlled, double-blind trial	VagiBIOM suppository vs. placebo	Improvement in vaginal pH, VAS itching score, total Nugent score, and vaginal health index	Effective in improving vaginal *Lactobacillus *diversity and overall vaginal health	Yes	Described and appropriate	Yes	Described and appropriate	Yes	5

Bias and Limitations

This systematic review faces several limitations that could affect its comprehensiveness and reliability. Potential selection bias exists, as relevant studies may have been inadvertently overlooked despite extensive search efforts. Limiting the search to English and Spanish publications also excluded studies in other languages, potentially narrowing the scope of the review. Publication bias is another concern, with studies showing positive results being more likely to be published, which can skew the findings and overestimate the effectiveness of probiotics.

The included studies exhibit considerable heterogeneity in probiotic strains, dosages, intervention durations, and population characteristics, complicating direct comparisons and generalizability. Variations in study design, sample size, and outcome measures further contribute to this inconsistency. Additionally, while the Jadad scale was used for quality assessment, it primarily focused on randomization, blinding, and withdrawals without thoroughly evaluating other crucial quality aspects such as allocation concealment, adherence to intervention protocols, and handling of missing data.

Most studies had short follow-up periods, insufficient to capture the long-term effects of probiotics on preventing vulvovaginal infections. Longer-term studies are necessary to assess sustained impacts. Confounding factors such as adherence to the probiotic regimen, concurrent medication use, and variations in diet and lifestyle were not consistently controlled, which could influence outcomes and limit attributing effects solely to the probiotic interventions.

Discussion

These studies collectively emphasize the potential advantages of probiotics in controlling BV and VVC, highlighting substantial improvements in clinical results and the restoration of the vaginal microbiota. Nonetheless, the results exhibited some inconsistencies in effectiveness, underscoring the necessity for additional exploration.

Several studies, including those by Mändar et al. [8] and Park et al. [11], have demonstrated that probiotics significantly improve BV and VVC symptoms and reduce recurrence rates. Mändar et al. [8] reported that probiotics, administered both orally and vaginally, resulted in significant symptom relief and an increase in *Lactobacillus *spp., supporting the therapeutic potential of probiotics in managing these infections effectively. Similarly, Park et al. [11] found that the MED-01 probiotic formulation significantly reduced the Nugent scores, indicating improved vaginal microbiota and effective BV treatment. In contrast, Yang et al. [9] and Jepsen et al. [12] reported no significant differences in vaginal microbiota diversity or improvement in symptoms with probiotic use compared with placebo. These discrepancies may stem from differences in the study populations, probiotic strains, dosages, and duration of treatment. The impact of probiotics on the restoration of a healthy vaginal microbiota has been highlighted in multiple studies. Ang et al. [10] and Koirala et al. [15] found that probiotics led to significant improvements in both the vaginal and gastrointestinal microbiota. Ang et al. [10] noted that probiotics prevented adverse microbiota changes during vaginal infections, while Koirala et al. [15] observed a reduction in the relative abundance of *Gardnerella *spp., a common pathogen associated with BV. These findings suggest that probiotics play a crucial role in maintaining vaginal health by promoting beneficial bacterial growth. Armstrong et al. [13] investigated the anti-inflammatory effects of the *Lactobacillus crispatus* CTV-05 probiotic, finding reduced concentrations of IL-1 alpha and soluble E-cadherin, which are markers of genital inflammation and epithelial barrier disruption. This study indicates that probiotics may not only address microbial imbalances but also enhance the integrity of the vaginal epithelial barrier, offering additional protective benefits.

Studies by Bangar et al. [14] and Vaccalluzzo et al. [16] confirmed the safety and feasibility of probiotics. Bangar et al. [14] reported that *Lactobacillus *vaginal tablets (VTs) were well-tolerated, although they did not show any added benefits over metronidazole in reducing BV recurrence. Vaccalluzzo et al. [16] highlighted the effectiveness of *Lacticaseibacillus rhamnosus* in restoring physiological pH and reducing pathogenic bacteria. Bacterial vaginosis and VVC endanger pregnant women by increasing the risk of preterm birth (PTB) and premature rupture of membranes (PROM) through adverse effects on the vaginal microbiota and host immunity. Bacterial vaginosis results from the overgrowth of anaerobic bacteria and a reduction in *Lactobacillus *species, which is crucial for a healthy vaginal environment. Pathogenic bacteria in BV cause inflammation and weaken fetal membranes, increasing the risk of PROM and PPROM. *Lactobacillus *species, including *L. acidophilus*, *L. fermentum*, *L. crispatus*, and *L. jensenii*, enhance vaginal health by producing lactic acid and hydrogen peroxide, which lower pH and inhibit pathogenic bacteria. A higher presence of *Lactobacillus *species in the vaginal microbiota correlates with a reduced risk of BV and urinary tract infections, both associated with adverse pregnancy outcomes such as PTB and PROM [17].

Vanda et al. [17] provide significant insights into oral probiotics' role in preventing complications like PPROM and PROM. This randomized placebo-controlled double-blind trial showed that administering oral probiotics significantly reduced the incidence of PPROM and PROM in pregnant women. These findings emphasize the potential of probiotics to maintain or restore vaginal health, which is crucial for preventing serious pregnancy-related complications. Probiotics positively influence the vaginal microbiome, potentially reducing the risk of infection and inflammation, leading to PPROM and PROM. This study highlights the importance of incorporating probiotics in prenatal care to enhance maternal and neonatal health outcomes. However, larger, long-term studies are necessary to validate these findings and establish comprehensive clinical guidelines for probiotic use.

Vivekanandan et al. [18] conducted a thorough study evaluating the *Lactobacillus *vaginal suppository's efficacy in enhancing vaginal health. This randomized, double-blind, placebo-controlled trial showed significant improvements in vaginal pH balance, reduced itching, improved Nugent scores, and overall improved vaginal health index scores in women using probiotics. These findings support the *Lactobacillus *suppository as an effective intervention for increasing the diversity and abundance of beneficial *Lactobacillus *species, which is vital for preventing and managing conditions such as BV and VVC. These results suggest that probiotic suppositories could be a valuable alternative or complement to conventional antimicrobial therapies, offering a non-antibiotic approach to maintaining vaginal health. Future research should compare different probiotic formulations and delivery methods to optimize clinical applications and enhance patient adherence and satisfaction.

Biases and Limitations

This systematic review encountered several constraints that affected its comprehensiveness and dependability. One of the limitations is the potential for selection bias, as pertinent studies may have been overlooked despite a thorough search. By limiting the search to English and Spanish publications, studies in other languages were excluded, which may have narrowed the scope of the review. Publication bias is also a concern, as studies with positive outcomes are more likely to be published, which could skew the findings and overestimate the effectiveness of probiotics. The studies included in the review displayed disparities in probiotic strains, dosages, duration of intervention, and characteristics of the study population, which complicates direct comparisons and generalizability. Variations in the study design, sample size, and outcome measures added to the inconsistency. The Jadad scale, which was employed for quality assessment, primarily focused on randomization, blinding, and withdrawals without providing a thorough evaluation of other quality aspects, such as allocation concealment, adherence to the intervention protocols, and handling of missing data. Most studies had short follow-up periods, which were insufficient for capturing the long-term effects of probiotics on preventing vulvovaginal infections, necessitating long-term studies to assess sustained impacts. Confounding factors, such as adherence to the probiotic regimen, concurrent medication use, and variations in diet and lifestyle, were not consistently controlled, which could have influenced the outcomes and limited the attribution of effects solely to probiotic interventions.

## Conclusions

The selected studies collectively underscore the potential benefits of probiotics in managing BV and VVC. The evidence indicates significant improvements in clinical outcomes and the restoration of the vaginal microbiota, although the efficacy varies. Probiotics have shown promise in alleviating symptoms, reducing recurrence rates, and promoting the growth of beneficial bacteria while reducing pathogenic species. They also appear to provide additional benefits, such as reducing genital inflammation and maintaining the integrity of the vaginal epithelial barrier.

Probiotics have been shown to be safe and well-tolerated, with some studies suggesting their use as a valuable alternative or adjunct to conventional antimicrobial therapies. Furthermore, the potential role of probiotics in prenatal care, particularly in preventing complications such as PPROM and PROM, highlights their broader clinical significance. Most studies included in this review have relatively short follow-up periods. Longer-term studies are essential to assess the sustained impact of probiotic interventions on preventing vulvovaginal infections. These studies should aim to evaluate the long-term safety, efficacy, and potential for recurrence reduction over extended periods, providing more comprehensive evidence for clinical practice.

However, the variability in study results highlights the need for further research. Future studies should focus on standardizing probiotic strains, dosages, and treatment durations and exploring different delivery methods to optimize clinical applications and enhance patient adherence and satisfaction. Establishing comprehensive clinical guidelines based on large-scale, long-term studies is crucial for integrating probiotics effectively into the management and prevention of BV and VVC. 

The evidence underscores the potential of probiotics in managing BV and VVC, with specific strains like *Lactobacillus rhamnosus* and* Lactobacillus paracasei *showing promise. Probiotics can alleviate symptoms, reduce recurrence rates, and promote beneficial bacterial growth while minimizing pathogenic species. Despite these positive outcomes, variability in study results necessitates the standardization of probiotic formulations, dosages, and treatment protocols. Long-term studies are crucial to validate these findings and establish comprehensive clinical guidelines, ensuring the effective integration of probiotics into the management and prevention of BV and VVC.
